# Comparison of the accuracy of 9 intraocular lens power calculation formulas after SMILE in Chinese myopic eyes

**DOI:** 10.1038/s41598-023-47990-0

**Published:** 2023-11-23

**Authors:** Liangpin Li, Liyun Yuan, Kun Yang, Yanan Wu, Simayilijiang Alafati, Xia Hua, Yan Wang, Xiaoyong Yuan

**Affiliations:** 1 Clinical College of Ophthalmology, Tianjin Medical University, Tianjin, 300020 China; 2 Tianjin Key Laboratory of Ophthalmology and Visual Science, Tianjin Eye Institute, Tianjin Eye Hospital, Tianjin, 300020 China; 3 School of Medicine, Nankai University, Tianjin, 300071 China; 4 Tianjin Aier Eye Hospital, Tianjin University, Tianjin, 300190 China

**Keywords:** Medical research, Clinical trial design

## Abstract

As of 2021, over 2.8 million small-incision lenticule extraction (SMILE) procedures have been performed in China. However, knowledge regarding the selection of intraocular lens (IOL) power calculation formula for post-SMILE cataract patients remains limited. This study included 52 eyes of 26 myopic patients from northern China who underwent SMILE at Tianjin Eye Hospital from September 2022 to February 2023 to investigate the suitability of multiple IOL calculation formulas in post-SMILE patients using a theoretical surgical model. We compared the postoperative results obtained from three artificial intelligence (AI)-based formulas and six conventional formulas provided by the American Society of Cataract and Refractive Surgery (ASCRS). These formulas were applied to calculate IOL power using both total keratometry (TK) and keratometry (K) values, and the results were compared to the preoperative results obtained from the Barrett Universal II (BUII) formula for the SMILE patients. Among the evaluated formulas, the results obtained from the Emmetropia Verifying Optical 2.0 Formula with TK (EVO-TK) (0.40 ± 0.29 D, range 0–1.23 D), Barrett True K with K formula (BTK-K, 0.41 ± 0.26 D, range 0.01–1.19 D), and Masket with K formula (Masket-K, 0.44 ± 0.33 D, range 0.02–1.39 D) demonstrated the closest proximity to BUII. Notably, the highest proportion of prediction errors within 0.5 D was observed with the BTK-K (71.15%), EVO-TK (69.23%), and Masket-K (67.31%), with the BTK-K showing a significantly higher proportion than the Masket-K (*p* < 0.001). Our research indicates that in post-SMILE patients, the EVO-TK, BTK-K, and Masket-K may yield more accurate calculation results. At their current stage in development, AI-based formulas do not demonstrate significant advantages over conventional formulas. However, the application of historical data can enhance the performance of these formulas.

## Introduction

Small incision lenticule extraction (SMILE) is the latest generation of refractive surgery and is widely utilized for treating refractive errors. SMILE involves creating a corneal lenticule within the stromal layer, which is subsequently removed through a small incision. This innovative technique eliminates the need for corneal flap preparations while ensuring enhanced precision and superior biomechanical outcomes compared to alternative methods^1^. Cataracts, one of the most common causes of blindness worldwide^2,3^, are very common in the elderly population, and studies have shown that their prevalence in the population over 80 years old is as high as 60% to 90%^4,5^. In the future, we can envision a substantial number of cataract patients will have previously undergone SMILE surgery. However, this presents a dilemma for surgeons. While these individuals hold high expectations for optimal postoperative visual quality, the corneal morphology undergoes changes because of the previous refractive surgery. Consequently, the accuracy and predictability of calculating the dioptres for intraocular lenses (IOLs) are significantly reduced. Even more challenging is the inability to rectify any refractive complications that may arise after the operation through additional laser surgery, as keratectomy has already been performed.

As one of the most crucial elements for ensuring the accurate calculation of IOL power, the IOL formula plays a pivotal role. Most conventional formulas predict the effective lens position (ELP) based on corneal curvature^6^, but the corneal curvature of the eye can change significantly after refractive surgery, leading to substantial errors^7^. To better suit various eye conditions, IOL formulas continue to undergo innovative changes, including approaches that no longer rely on corneal curvature for calculating the ELP, the utilization of the double-K method, and the integration of artificial intelligence (AI) methods introduced in recent years^8–11^. As a relatively new method in refractive surgery, we cannot directly apply previous data from Laser-assisted In Situ Keratomileusis (LASIK) to the assessment of cataract patients with a history of SMILE. Consequently, there is an urgent need to identify more accurate formulas for this specific patient group. However, due to the limited history of SMILE surgery and the fact that most patients are young, obtaining a sufficiently large sample of cataract patients who have undergone SMILE surgery has proven challenging, making it difficult to identify the most suitable formula directly. To address this issue, the theoretical surgical model proposed by Lazaridis, A. et al. currently stands as the most reliable approach in such research^12,13^. This theoretical model entails optical biometry measurements conducted before and after SMILE surgery, with the same formula used for both pre- and postoperative measurements. This approach allows for the evaluation of formula stability under various conditions, rendering it an ideal research method given the current circumstances. In this study, we adopted and refined this method to assess the accuracy of nine distinct formulas. These formulas encompass those developed for LASIK surgery and more recent AI-based formulas introduced in recent years.

## Results

This study included a total of 26 patients (52 eyes) who received treatment at Tianjin Eye Hospital in Tianjin, China, from September 2022 to February 2023. The mean follow-up period after SMILE surgery was 4 ± 2 months (ranging from 3 to 8 months). All patients underwent SMILE surgery for myopia correction, and their postoperative visual acuity was 20/20 or better. The preoperative mean K value was 43.36 ± 1.29, and the mean TK was 43.37 ± 1.30. After SMILE surgery, the mean K and TK values were 39.58 ± 1.47 and 38.85 ± 2.00, respectively. Further patient characteristics and biometric data were summarized in Table 1.

**Table 1:** Patient demographic and ocular biometry data before and after SMILE.

Eyes/patients	52/26		
Sex	M/F, 11/15		
Age (years)	26.19	(18, 25)	
AL (mm)			
	Preop	25.63 ± 0.81, (23.97, 27.53)	
	Postop	25.52 ± 0.81, (23.86, 27.44)	
ACD (mm)			
	Preop	3.81 ± 0.31	
	Postop	3.70 ± 0.28	
Keratometry (D)		K	TK
	Preop	43.36 ± 1.29	43.37 ± 1.30
	Postop	39.58 ± 1.47	38.85 ± 2.00
SE (D)			
	Preop	− 4.75	
	Postop	− 0.53	
CCT (μm)			
	Preop	548.11 ± 32.28	
	Postop	475.25 ± 40.02	

M = Male; F = Female; AL = Axial length; ACD = Anterior chamber depth; Preop = Preoperative; Postop = Postoperative; SE = Spherical equivalent; CCT = Central corneal thickness.

Using one-way ANOVA to analyse the variance of 18 different data points, we found a significant difference in PE and AE among the groups (*p* < 0.001). The one-sample t test comparing PE with zero revealed that, except for those of the EVO-TK (*p* = 0.951), Masket-TK (*p* = 0.885), BTK-TK (*p* = 0.059), and BTKNH-TK (*p* = 0.625), which demonstrated good consistency with the results of the BUII, the formula results were significantly different from zero (all *p* < 0.001).

**Figure 1:**
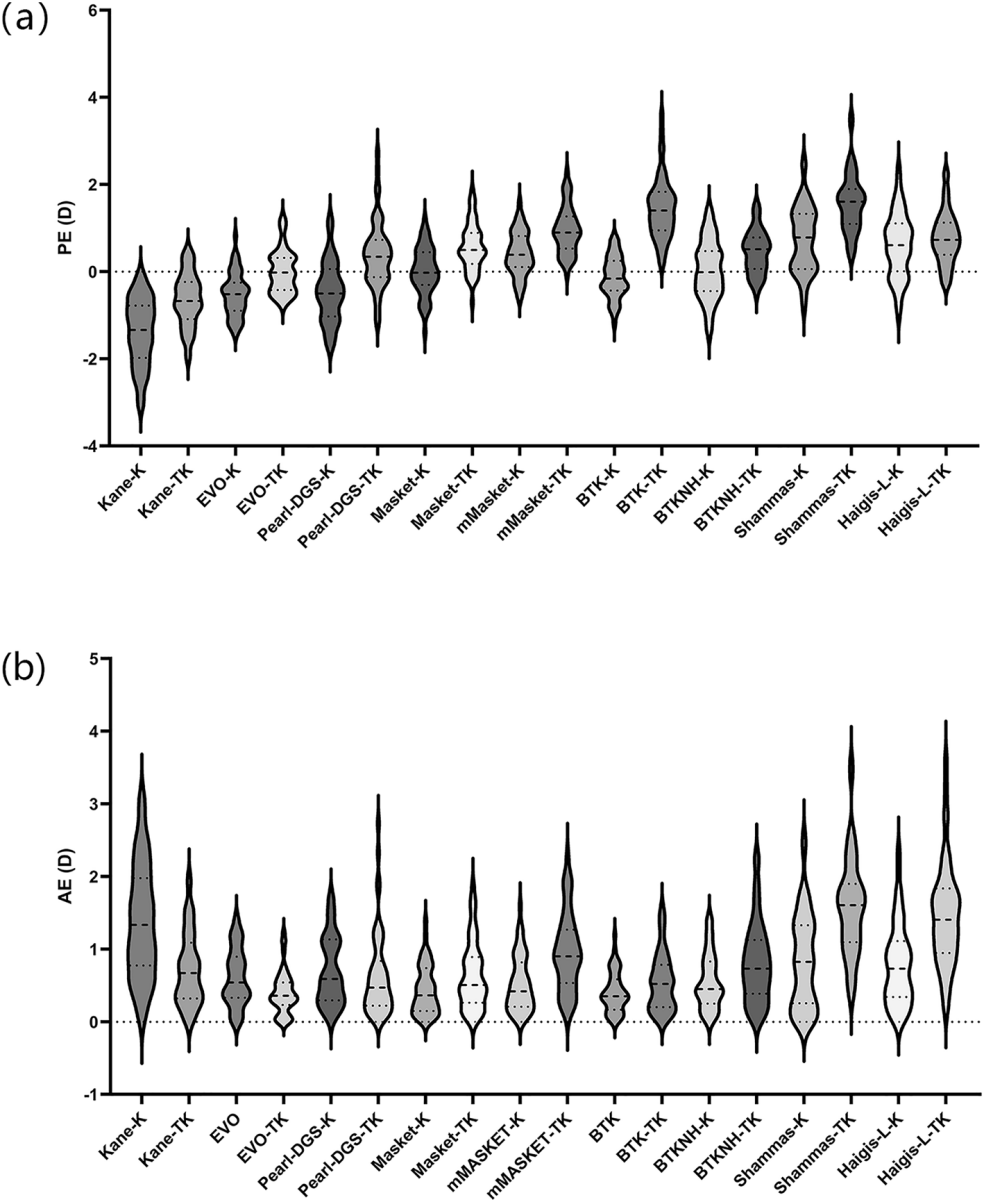
(**a**) Violin plot of the prediction errors (PE) of 9 formulas. The formulas with the smallest PEs were Kane-K (− 1.373 ± 0.78 D), Kane-TK (− 0.64 ± 0.61 D), and EVO-K (− 0.53 ± 0.50 D), and the formulas with the largest PEs were Shammas-TK (1.58 ± 0.68 D), BTK-TK (1.46 ± 0.71 D), and m-Masket-TK (0.96 ± 0.54 D). (**b**) Violin plot of the absolute prediction errors (AE) of 9 formulas. The formulas with the lowest AEs were EVO-TK (0.40 ± 0.29, range 0–1.23, MedAE = 0.36 D), BTK-K (0.41 ± 0.26, range 0.01–1.19, MedAE = 0.35), and Masket-K (0.44 ± 0.33, range 0.02–1.39, MedAE = 0.37).

The formulas with the lowest PE were the Kane-K (− 1.373 ± 0.78 D), Kane-TK (− 0.64 ± 0.61 D), and EVO-K (− 0.53 ± 0.50 D), suggesting a tendency for a hyperopic shift. The formulas with the highest PE were the Shammas-TK (1.58 ± 0.68 D), BTK-TK (1.46 ± 0.71 D), and m-Masket-TK (0.96 ± 0.54 D), indicating a tendency for a myopic shift postoperatively (Fig. 1a).

Figure 1b illustrates violin plots depicting the distribution of each formula's absolute error (AE). The formulas with an average AE of less than 0.5 D were the EVO-TK (0.40 ± 0.29, range 0 to 1.23, MedAE = 0.36 D), BTK-K (0.41 ± 0.26, range 0.01–1.19, MedAE = 0.35), and Masket-K (0.44 ± 0.33, range 0.02–1.39, MedAE = 0.37). Tukey's HSD showed no significant pairwise differences among the formulas (EVO-TK: BTK-K, *p* = 0.690; EVO-TK: Masket-K, *p* = 0.594; BTK-K: Masket-K, *p* = 0.648). The formulas with an average AE exceeding 1 D were Shammas-TK (1.584 ± 0.68, range 0.33–3.56, MedAE = 1.61), Haigis-L-TK (1.464 ± 0.71, range 0.2–3.58, MedAE = 1.41), and Kane-K (1.373 ± 0.78, range 0.08–3.03, MedAE = 1.34), and Tukey's HSD showed significant differences among all three pairs (all *p* < 0.001). More detailed information can be found in Table 2.

**Table 2:** Preoperative refractive absolute prediction errors relative to the BUII in 52 eyes.

Formula	Mean (D)	SD (D)	Range (D)	Median (D)
EVO-TK	0.40	0.29	0.00 to 1.23	0.36
BTK-K	0.41	0.28	0.01 to 1.19	0.35
Masket-K	0.44	0.33	0.02 to 1.39	0.37
m-Masket-K	0.53	0.38	0.01 to 1.59	0.42
BTK-TK	0.55	0.40	0.02 to 1.57	0.52
BTKNH-K	0.56	0.38	0.01 to 1.42	0.45
EVO-K	0.61	0.39	0.01 to 1.41	0.54
MASKET-TK	0.64	0.46	0.03 to 1.86	0.51
Pearl-DGS-TK	0.64	0.59	0.04 to 2.73	0.47
Pearl-DGS-K	0.70	0.47	0.02 to 1.71	0.59
Kane-TK	0.72	0.50	0.01 to 1.96	0.67
Haigis-L-K	0.78	0.56	0.01 to 2.35	0.73
BTKNH-TK	0.81	0.55	0.04 to 2.26	0.73
Shammas-K	0.83	0.65	0.00 to 2.51	0.83
m-Masket-TK	0.96	0.54	0.06 to 2.28	0.90
Kane-K	1.37	0.78	0.08 to 3.03	1.34
Haigis-L-TK	1.46	0.71	0.20 to 3.58	1.41
Shammas-TK	1.58	0.68	0.33 to 3.56	1.61

EVO, Emmetropia Verifying Optical 2.0; BTK, Barrett True K; BTKNH, Barrett True K No History; m-Masket, modified-Masket; K, keratometry and TK, total keratometry; D, dioptre.

**Figure 2:**
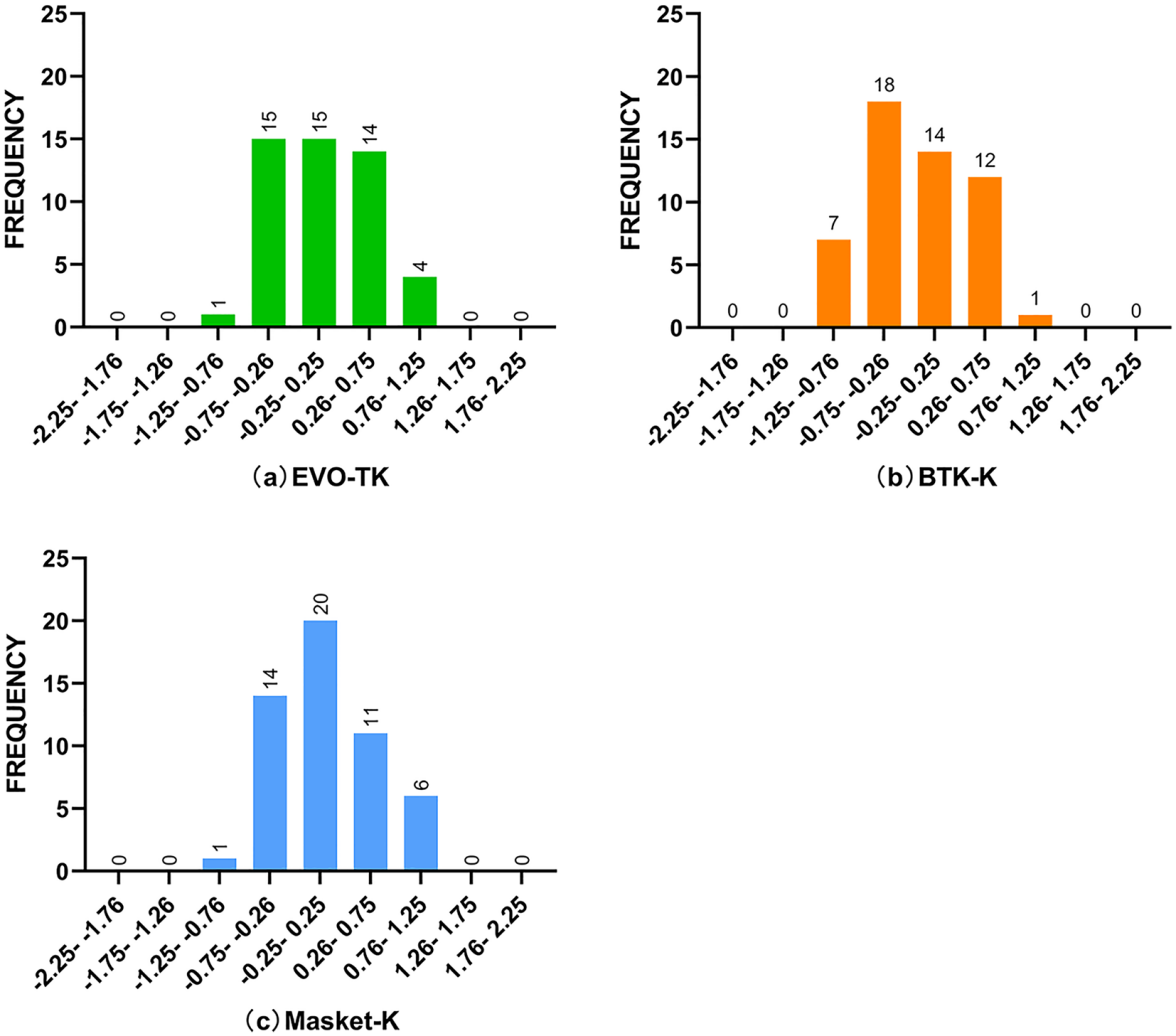
More detailed frequency profiles of the EVO-TK, BTK-K, and Masket-K formulas.

Figure 2 presents a more detailed frequency distribution of the PE in these three formulas. Among them, Masket-K demonstrates the highest distribution between − 0.25 D and 0.25 D, with a total of 20 eyes. EVO-TK shows a uniform distribution on both sides of 0 D. On the other hand, BTK-K is predominantly distributed between − 0.75 D and − 0.25 D, indicating slightly lower results compared to BUII, which may result in mild hyperopic shift.

In addition, when using TK values for calculations, Tukey's HSD test for AE demonstrated that the inclusion of TK was superior to the inclusion of K values in all three artificial intelligence formulas. There were significant differences for the Kane and EVO formulas, while no significant difference was found with the Pearl-DGS formula (Kane: *p* < 0.001, EVO: *p* = 0.008, Pearl-DGS: *p* = 0.961). The remaining six formulas showed the opposite trend, where the accuracy significantly decreased when TK values were used; the differences were not significant for the m-Masket and Shammas formulas (Masket *p* < 0.001, m-Masket *p* = 0.999, BTK *p* < 0.001, BTKNH *p* < 0.001, Shammas *p* = 0.299, Haigis-L *p* < 0.001).

**Figure 3:**
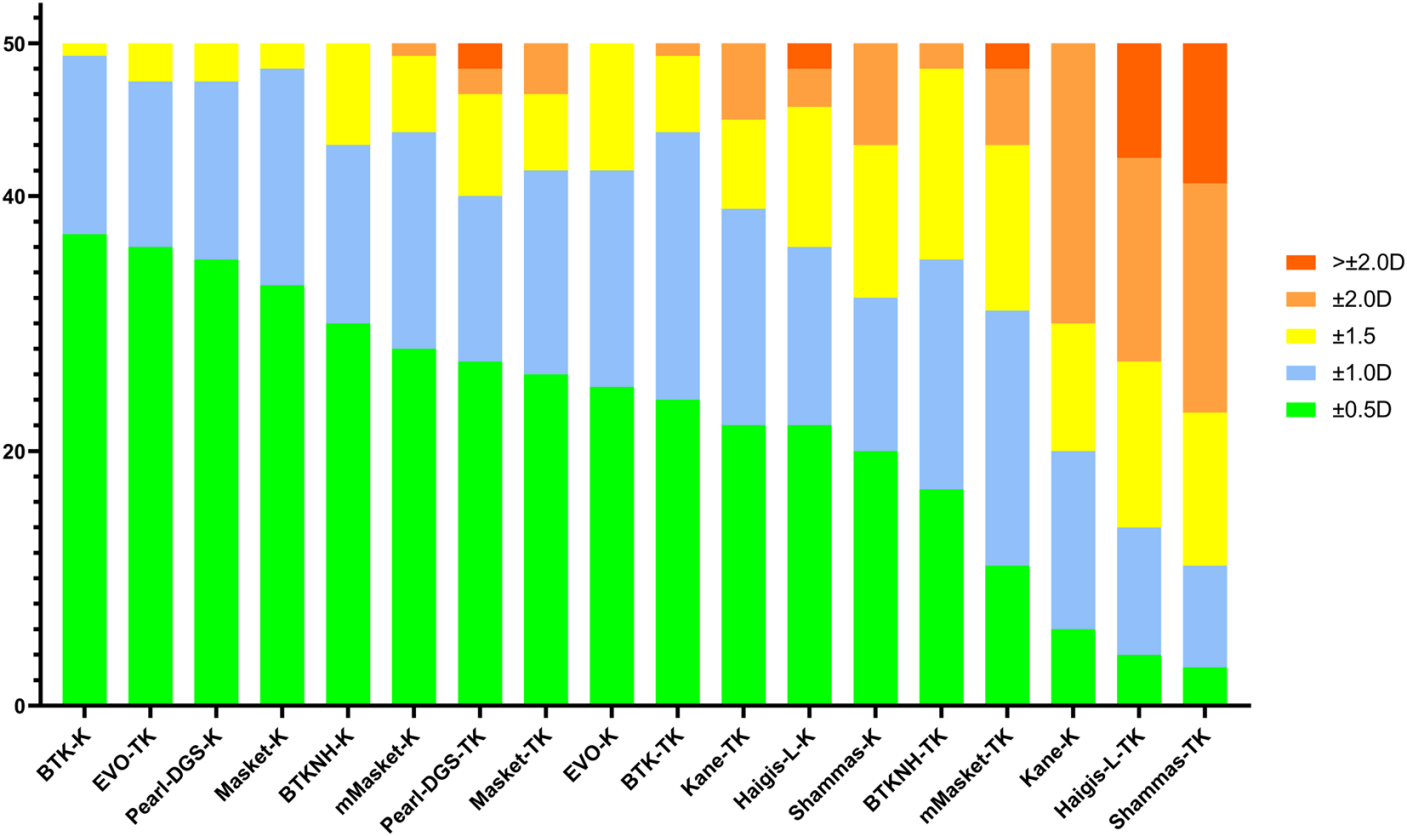
Stacked histogram analysis comparing the percentage of eyes within given prediction error ranges relative to the preoperative BUII. The formulas were sorted by the proportion of eyes within ± 0.50 D in descending order. The formulas with the highest proportion of AEs < 0.5D were the BTK-K (71.15%), EVO-TK (69.23%), and Masket-K (67.31%).

The distribution of AE within different ranges (< 0.5 D, 0.5 D-1.0 D, 1 D-2 D, and > 2 D) is shown in Table 3, and Fig. 3 is the stacked histogram of these formulas. Among them, the formulas with the highest proportion of AE < 0.5 D were BTK-K (71.15%), EVO-TK (69.23%), and Masket-K (67.31%). A more detailed frequency distribution of the EVO-TK, BTK-K, and Masket-K formulas is shown in Fig. 3. The chi-square test showed that the proportion of AE within 0.5 D for the BTK-K formula was significantly higher than that for the Masket-K formula (χ^2^ = 22.845, *p* < 0.001). There were no significant differences between BTK-K and EVO-TK or between EVO-TK and Masket-K (χ^2^ = 1.278, *p* = 1.000; × 2 = 0.93, *p* = 1.000, respectively). The formulas with the highest proportion of AE within 1.0 D were BTK-K (98.08%), Masket-K (96.15%), and EVO-TK (94.23%). The chi-square test showed no significant differences among the three formulas (BTK-K and Masket-K: χ^2^ = 0.41, *p* = 1.000; BTK-K and EVO-K: χ^2^ = 0.62, *p* = 1.000; Masket-K and EVO-TK: χ^2^ = 1.27, *p* = 1.000). The formulas with a AE greater than 2 D were the Pearl-DGS-TK (3.84%), Haigis-L-K (3.84%), m-Masket-TK (3.84%), Haigis-L-TK (17.30%), and Shammas-TK (21.15%), indicating that using these formulas may result in larger errors.

**Table 3:** Percentages of eyes falling within different ranges of AE among the formulas.

Formula	± 0–0.5 D (%)	± 0.5–1.0 D (%)	± 1.0–1.5 D (%)	± 1.5–2.0 D (%)	> ± 2.0 D (%)
BTK-K	71.15	26.92	1.92	0.00	0.00
EVO-TK	69.23	25.00	5.77	0.00	0.00
Masket-K	63.46	32.69	3.85	0.00	0.00
BTKNH-K	57.69	26.92	15.38	0.00	0.00
m-Masket-K	53.85	32.69	11.54	1.92	0.00
Pearl-DGS-TK	51.92	25.00	15.38	3.85	3.85
Masket-TK	50.00	30.77	11.54	7.69	0.00
EVO-K	48.08	32.69	19.23	0.00	0.00
BTK-TK	46.15	40.38	11.54	1.92	0.00
Haigis-L-K	42.31	26.92	21.15	5.77	3.85
Pearl-DGS-K	42.31	25.00	32.69	0.00	0.00
Kane-K	42.31	32.69	13.46	11.54	0.00
Shammas-K	38.46	23.08	23.08	15.38	0.00
BTK-NO-TK	32.69	34.62	28.85	3.85	0.00
m-Masket-TK	21.15	38.46	25.00	11.54	3.85
Kane-TK	11.54	26.92	19.23	42.31	0.00
Haigis-L-TK	7.69	19.23	25.00	30.77	17.31
Shammas-TK	5.77	15.38	23.08%	34.62	21.15

AE: absolute prediction errors EVO; Emmetropia Verifying Optical 2.0; BTK, Barrett True K; BTKNH, Barrett True K No History; m-Masket, modified-Masket; K, keratometry and TK, total keratometry; D, dioptre.

## Discussion

While SMILE surgery has become a mainstream refractive procedure, there remains a lack of comprehensive knowledge regarding accurate IOL power calculation for cataract patients who have previously undergone SMILE surgery. Currently, most formulas designed for postrefractive surgery cases are primarily based on data from LASIK or photo refractory keratectomy (PRK) procedures^14–17^. Additionally, it is worth noting that commonly used measurement devices often lack specific options for post-SMILE patients. Given the limited number of patients with a history of both SMILE surgery and cataracts, our study relies on a theoretical surgical model to investigate the performance of various IOL power calculation formulas. In our research, we observed that the BTK-K, Masket-K, and EVO-TK formulas closely approximated the preoperative refractive outcomes achieved with the BUII formula. Remarkably, all three formulas demonstrated an average prediction error (PE) of less than 0.5 D. These formulas show promise in optimizing the refractive outcomes for this specific patient group, reducing the potential for residual refractive errors and enhancing the overall success of cataract surgery in SMILE patients.

The BTK formula, developed based on BUII, has demonstrated outstanding performance in numerous retrospective consecutive case series studies. Similar to BUII, the specific calculation method for BTK has not been publicly disclosed. However, its accuracy in calculating IOL power after refractive surgery has been validated by multiple research institutions. Ferguson et al.^18^ reported that BTK outperformed other ASCRS formulas in both myopic and hyperopic postlaser refractive surgery patients (96 postmyopic eyes and 47 posthyperopic eyes). In both postmyopic and posthyperopic patients, the BTK formula had the highest percentage of errors within ± 0.25 D (44.8% and 42.6%, respectively), and the average error was less than 0.5 D (0.41 D). Abulafia et al.^19^ found that the BTK had significantly smaller prediction errors and a higher percentage of eyes within ± 0.50 D than the Shammas and Haigis-L formulas in postmyopic LASIK cases (88 postmyopic eyes). Notably, Savini et al.^20^ found that when combined with measured posterior corneal power (PK, obtained with a Pentacam) in cases with available historical data, the BTK yielded the best results (lowest prediction error of 0.52 D and the highest percentage of eyes with a prediction error within ± 0.25 D [54%]). However, using PK without historical data led to larger errors (50 postmyopic eyes). In our study, we found that the BTK also achieved satisfactory results in patients after SMILE surgery, with an average prediction error of less than 0.5 D, over 70% of eyes having a prediction error within 0.5 D, and only 1.92% of eyes having a prediction error greater than 1 D.

Similarly, the Masket formula has gained wide recognition for its accuracy in post-LASIK patients, and several studies have demonstrated a performance on par with the BTK formula^21–23^. Chen et al.'s meta-analysis showed that the Masket formula was more accurate than the Haigis formula in patients who had undergone refractive surgery^24^. The Masket formula, specifically designed for refractive surgery, refines the final IOL power using the following equation: IOLpost + (RC × 0.326) + 0.101 (where IOLpost is the calculated IOL power following refractive surgery, and RC represents the refractive change at the corneal plane)^25^. Savini et al.'s prospective study suggested that the Masket formula might be the most reliable choice for postrefractive surgery cases that lack preoperative keratometry data but have known refractive changes^22^. Undoubtedly, while this formula's principle is straightforward, it has demonstrated strong performance. Moreover, it offers a unique advantage in cases where only preoperative manifest refraction data are available and corneal curvature data are missing. However, the Masket formula has shortcomings, notably its unsuitability for patients lacking preoperative manifest refraction data. This limitation might explain why the formula is not as widely adopted and researched as formulas such as BTK.

Among the three formulas, EVO-TK, the only AI-based formula supported by a large dataset, also yielded satisfactory results. Consistent with this, previous studies have demonstrated excellent performance of the EVO formula in cases with various corneal abnormalities. Ferrara et al.^26^ found that the EVO formula showed higher accuracy in post-RK cataract patients than the SRK/T and BTK formulas in a study involving 27 patients. In another retrospective study of 110 patients with congenital cataracts, Lin et al.^27^ found that the EVO formula performed best in children, especially those under 24 months of age and with an axial length less than 21 mm. In a recent study that incorporated the EVO formula, the mean absolute error for the EVO formula was 0.68 D (302 eyes of 302 patients, postmyopic refractive surgery)^16^. Therefore, we believe that using TK values when applying the EVO formula for postrefractive surgery patients may lead to better results. Although the EVO formula has a dedicated module for postrefractive surgery cases, there is currently a lack of research on using the EVO formula to calculate refractive outcomes in such patients. As a relatively new formula, we hope to see more research support for its use in the future.

These three formulas all utilize historical data and include preoperative spherical equivalent (SE) and postoperative SE as parameters to correct the calculation results, emphasizing the value of accurate historical data in calculations for postrefractive surgery patients. Several studies have shown that the application of historical data improves calculation accuracy in patients after LASIK^20,28^. However, the limitation lies in the difficulty of obtaining such data due to the long intervals between cataract surgery and refractive surgery. It is not feasible to rely on every patient to keep their own medical records. We believe that storing standardized, preoperative and postoperative patient examination data in an online database would provide the greatest benefit to the patients^29^.

In this study, we found that the Kane and EVO formulas performed better when using TK, while the BTK, BTKNH, Masket, and Haigis should use K instead of TK. The IOL Master 700 calculates K using the traditional telecentric keratometry method, while TK is derived from swept-source OCT measurements of the posterior corneal surface combined with K values rather than being a direct measurement. Theoretically, TK should be more accurate and closer to the actual corneal power. However, some studies suggest that simply incorporating TK values may not be suitable for all formulas^30^. In a clinical retrospective comparative study, Danjo et al. found that in cases of single-focus IOL implantation (225 eyes of 225 patients, IOL Master 700), K provided better accuracy than TK for various axial lengths in routine cataract patients when using formulas such as the BUII, Haigis, SRK/T, Holladay 2, and Hoffer Q^31^. Chung et al.^32^ also found that in the selection of multifocal IOLs (543 eyes of 543 patients, IOL Master 700), K should be used instead of TK with formulas such as the BUII, Haigis, SRK/T, and Holladay 2. It appears that TK does not provide an advantage in routine cataract patients. However, in the selection of toric IOLs, combining TK with the Barrett toric formula can reduce the error in predicted residual astigmatism (247 eyes of 180 patients, IOL Master 700)^33^. Additionally, Yeo et al.^34^ found in a study of postrefractive surgery patients (64 eyes of 49 patients, IOL Master 700) that formulas such as the BTK, Haigis-L, Shammas-PL, EVO, Hoffer Q, Holladay I, and SRK/T yielded better results when TK was used. Similarly, studies have demonstrated that TK is superior to K in cases with complex corneal conditions such as combined corneal endothelial diseases and keratoconus^35,36^. Therefore, in designing this study, we used the BUII with K preoperatively and compared TK and K postoperatively. We found that except for the EVO and Kane formulas, which performed better when using TK, the formulas had larger errors. Further research is needed to determine whether TK should be used in postrefractive surgery patients.

Although there are currently no specific formulas or options developed exclusively for SMILE, it appears that we can still make use of various formulas designed for post-LASIK calculations as alternatives. Significantly, it is imperative to acknowledge that any formula can exhibit deviations. Some formulas may incline towards yielding outcomes associated with hyperopic shift, while others may exhibit a propensity for outcomes indicative of myopic shift. Moreover, the influence of individual surgeon-specific factors must not be underestimated. Consequently, this underscores the advantage of employing multiple formulas as points of reference in our calculations. Achieving uniform results from different formulas provides us with confidence in selecting the IOL power and reducing the rate of significant refractive surprises.

## Methods

This research project was approved by the Ethics Committee of Tianjin Eye Hospital and was conducted according to the principles of the Helsinki Declaration. The study included 52 eyes of 26 patients with myopia who received refractive treatment at Tianjin Eye Hospital from October 2022 to March 2023. Each patient was informed of the research content and signed an informed consent form before the surgery.

### Patient data collection

All patients were evaluated by ophthalmology before the operation, including uncorrected distance visual acuity and corrected distance visual acuity, manifest refraction, cycloplegic refraction, slit lamp examination, and optical biometrics with an IOL Master700 (Carl Zeiss Meditec AG, Jena, Germany) and corneal tomography with a Pentacam HR (OCOLUS Optigerate GmBH). Because of the cornea's biomechanical properties and the patient's refractive status stabilizing three months postsurgery, a postoperative evaluation was conducted, including assessments of uncorrected distance visual acuity, corrected distance visual acuity, manifest refraction, and IOLMaster 700 measurements, at a minimum of three months after the surgical procedure^37–39^.

### Inclusion and exclusion criteria

The inclusion criteria included age more than 18 years, stable refraction 2 years before the operation, corrected distance visual acuity of 20/20 or better, suspended use of soft contact lenses for more than 2 weeks, and suspended use of hard contact lenses for more than 4 weeks. The exclusion criteria were slit lamp examination for any corneal or lens opacity or pathological changes, previous corneal surgery, ocular trauma or intraocular surgery, severe dry eye, glaucoma, corneal disease or ocular infection, keratoconus or suspected keratoconus, remaining stromal expected < 280 µm, posterior scleral staphyloma and so on.

### Surgical procedure

All operations were performed by the same experienced surgeon (Wang Y) using the VisuMax femtosecond laser (Carl Zeiss Meditec AG, Jena, Germany). There were no intraoperative or postoperative complications.

### IOL power calculation

Since the lens and the posterior segment of the eye are almost unchanged except for the change in the cornea caused by the SMILE procedure, the change in IOL power before and after the operation should theoretically be equal to the change in the corneal refractive power caused by the operation. In this study, a theoretical surgical approach simulating lens removal was adopted^13^. Preoperative and postoperative optical biometry measurements were performed using the IOL Master 700. Preoperatively, we used the BUII formula to calculate IOL power (the target refraction was selected as the equivalent spherical dioptre of the patient before SMILE; for example, if the preoperative SE was − 6 D and the postoperative SE was − 0.5 D, then the calculation would be as follows: − 6 D-(− 0.5 D) = − 5.5 D. While IOL powers are typically produced in 0.5 D increments as the smallest unit, for the purpose of facilitating subsequent calculations, we opted for the precise recommended power of − 5.5 D to ensure greater accuracy) Postoperatively, three different AI-based formulas, namely, the Kane, EVO, and Pearl-DGS, along with six commonly used formulas, namely, the BTK, BTKNH, Masket, m-Masket, Shammas, and Haigis-L, were employed to calculate the IOL power corresponding to a target refractive power of 0 D. Since some formulas do not provide the exact IOL power values for the target refraction, Microsoft Excel 2016 (Microsoft Corp.) was used to perform function calculations to obtain precise values. The availability of formulas and the IOL constants are summarized in Table 4. All calculation results were rounded to two decimal places. Each formula's calculation result minus the preoperative BUII calculation result yielded the PE, its absolute value represented the AE, and the median of the AE was abbreviated MedAE. During the calculations, the K and TK values were input into each formula separately, denoted as Kane-K/Kane-TK, BTK-K/BTK-TK, and so on. To maintain consistency among the formulas for IOL power calculation, the same model of IOL (AR40e, Johnson & Johnson Vision Care, Inc., A constant = 118.71; a0 = − 2.420, a1 = 0.157, a2 = 0.288) was chosen.

**Table 4:** Formulas selected and the A constant used in the calculations.

Formula	Website	A constant
Barrett Universal II	https://calc.apacrs.org/barrett_universal2105/	118.71
Barrett True K	https://ascrs.org/tools/post-refractive-iol-calculator	118.71
Barrett True K No History	https://ascrs.org/tools/post-refractive-iol-calculator	118.71
Masket	https://ascrs.org/tools/post-refractive-iol-calculator	118.71
Modified-Masket	https://ascrs.org/tools/post-refractive-iol-calculator	118.71
Shammas	https://ascrs.org/tools/post-refractive-iol-calculator	118.71
Haigis-L	https://ascrs.org/tools/post-refractive-iol-calculator	a0 = − 2.420 a1 = 0.157 a2 = 0.288
Pearl-DGS	https://iolsolver.com/complex	118.71
Emmetropia Verifying Optical 2.0	https://www.evoiolcalculator.com/calculator.aspx	118.71
Kane	https://www.iolformula.com/	118.71

### Statistical analysis

Descriptive statistics, including the mean, standard deviation (SD), and range, were calculated using Microsoft Excel 2016 (Microsoft Corp.). The statistical analysis was conducted using SPSS 26.0 software (IBM Corp.; Armonk, NY, USA). The Kolmogorov‒Smirnov test was used to assess the normality of the distribution of the results of each calculation method, specifically for PE and AE. The results are expressed as the mean ± standard deviation. A one-sample t test was used to compare the differences in PE between various calculation methods and zero. One-way analysis of variance (ANOVA) was used to compare the differences in PE and AE among the different calculation methods. Post hoc tests were performed following ANOVA to identify specific group differences. To compare the differences in PE and AE among the different calculation methods, Tukey's honest significant difference (HSD) method was used for post hoc pairwise comparisons following ANOVA. The chi-square test was employed to compare the differences in the percentage of eyes falling within ± 0.5 D and ± 1.00 D of the AE among the formulas. Bonferroni correction was applied for multiple comparisons. A p value of less than 0.05 was considered statistically significant.

### Ethics approval and consent to participate

This study was approved by the ethics committee of Tianjin Eye Hospital (NO. KY-2023035) and performed in accordance with the tenets of the Declaration of Helsinki. Written informed consent was obtained from all participants.

## Data Availability

All data generated or analysed during this study are included in this published article and its supplementary information files.
